# Alcoholic and Nonalcoholic Liver Disease: Diagnostic Assessment and Therapeutic Perspectives

**DOI:** 10.1155/2019/8691502

**Published:** 2019-07-09

**Authors:** Stefano Gitto, Florian Bihl, Filippo Schepis, Fabio Caputo, Marina Berenguer

**Affiliations:** ^1^Department of Experimental and Clinical Medicine, Univeristy of Florence, Italy; ^2^University Hospital of Geneva, Geneva, Switzerland; ^3^University of Modena and Reggio Emilia, Modena, Italy; ^4^Unit of Internal Medicine, Department of Internal Medicine, SS Annunziata Hospital, Cento, Ferrara, Italy; ^5^“G. Fontana” Centre for the Study and Multidisciplinary Treatment of Alcohol Addiction, Department of Medical and Surgical Sciences, University of Bologna, Bologna, Italy; ^6^University of Valencia, Hospital La Fe, Valencia, Spain

Alcoholic liver disease (ALD) and nonalcoholic fatty liver disease (NAFLD) represent main causes of chronic liver disorder in the Western countries. Both ALD and NAFLD include a wide spectrum of conditions ranging from “simple” steatosis to steatohepatitis, cirrhosis, and hepatocellular carcinoma. ALD and NAFLD, besides sharing many histological patterns, have in common a negative impact on both cardiovascular and cancer risk. Consequently, ALD and NAFLD should be considered not only liver diseases but also systemic conditions that harmfully influence both morbidity and mortality.

ALD is caused by acute and/or chronic alcohol intake while NAFLD is outlined as the presence of hepatic steatosis without other causes of secondary fat accumulation such as noteworthy alcohol consumption and hereditary disorders. NAFLD is a complex illness with genetic and environmental risk factors and is typically coupled with metabolic conditions such as obesity and diabetes.

In this special issue, we present original research as well as review articles on the following topics: relationship between gut-liver axis and NAFLD, diagnostic ability of transient elastography in NAFLD (and simultaneous hepatitis B virus), noninvasive markers of fibrosis in ALD, NAFLD, and pancreas damage, role of traditional Chinese Medicine in the treatment of NAFLD, and use of antifibrotic drug for the cure of liver disease.

Recent advances demonstrated that microbiota shows a key role in many kinds of diseases. In particular, microbiota dysbiosis seems to be involved in the pathogenesis of nonalcoholic steatohepatitis (NASH) that definitely represents the progressive form of NAFLD. Modifications of microbiota-derived mediators, alterations of gut endothelial barrier, and translocation of inflammation mediators could negatively influence the outcome of liver damage due to NASH. G. Aragonès et al., with a whole review article, discuss the role of gut microbiota-derived mediators as potential diagnostic markers of NAFLD and NASH. The article underlines the relevance of developing noninvasive tools for the screening and diagnosis of NAFLD since the current gold standard (liver biopsy) shows some well-known limits related to safety, compliance, and feasibility. In particular, liver biopsy can lead to complications such as bleeding, pain, bile peritonitis, kidney puncture, or death. Furthermore, sampling errors are common due to effort with obtaining liver specimen representative of the entire organ. In the present special issue, other data about noninvasive diagnosis of NAFLD are reported. Specifically, the concordance between transient elastography (TE) and ultrasonography (US) in assessing liver fibrosis in patients with both chronic hepatitis B (CHB) and NAFLD is evaluated. Using the liver biopsy as standard comparison, G. Zhang et al. demonstrated that TE and US scores knowingly correlated with histologically proven liver fibrosis.

Obviously, also in patients with ALD to develop noninvasive methods of diagnosis would be important. L. Chrostek et al. analyzed the diagnostic values of the following noninvasive indirect markers of liver fibrosis: APRI, GAPRI, Forn's, FIB-4, Age-Platelet, and Hepascore in patients with ALD. Authors suggested that Hepascore showed lower diagnostic value in alcoholics than markers involving only liver enzymes, platelet count, and cholesterol. Remarkably, Forn's index emerged as the best marker among those analyzed.

The relationship between NAFLD and systemic damage is widely known particularly regarding cardiovascular risk. However, few data are available about the pathological relationship between NAFLD and pancreatitis. Abdominal obesity represents a chief element in the NAFLD pathogenesis and, at the same time, it can be a risk factor for acute pancreatitis. Interestingly, D. Wu et al. reported that NAFLD could exacerbate pancreatitis through releasing a large number of inflammatory factors. Notably, Kupffer cells (the resident macrophages of the liver) primarily mediate the NAFLD-related chronic inflammatory process.

The complexity of NAFLD and NASH pathogenesis explains the difficulties of scientific community in the development of widely approved effective treatment. The presence of parallel “hit” explains the systemic impact of NAFLD and the opportunity to develop drugs that can act on multiple levels. Y. Feng et al., conceptually in agreement with this concept, developed an animal study with the use of Jianpi Huoxue (JPHX) as treatment of NAFLD. The proposed Chinese herbal formula holds active compounds that regulate lipid metabolism and shows anti-inflammatory properties. JPHX exhibited hepatoprotective effects in animals with severe livery injuries (steatosis, inflammation, and fibrosis) decreasing lipid accumulation, inflammation, apoptosis, and fibrosis.

The main limitation of the available studies describing the treatment options for the treatment of NAFLD is the lack of a relevant effect on the fibrosis. In this direction, M. M. Arafah et al. planned a study using a rat model of liver fibrosis (obtained with intraperitoneal injections of carbon tetrachloride). A total of 45 rats were divided into 3 groups: control group, group II that received carbon tetrachloride for 8 weeks, and group III that was treated with carbon tetrachloride and nAG protein for the same time period. At the end of the experiment, serum levels of hyaluronic acid, PDGF-AB, TIMP-1, laminin, Procollagen III- N terminal peptide, and collagen IV alpha 1 chain were tested and liver biopsies were performed. NAG treatment decreased serum levels of the analyzed markers of fibrosis reducing also the histological fibrosis.

We believe that these articles may contribute to improve our knowledge in ALD and NAFLD that definitely represent the future of hepatology.

