# Corrigendum to “Alcoholic and Nonalcoholic Liver Disease: Diagnostic Assessment and Therapeutic Perspectives”

**DOI:** 10.1155/2020/4305190

**Published:** 2020-10-30

**Authors:** Stefano Gitt, Florian Bihl, Filippo Schepis, Fabio Caputo, Marina Berenguer

**Affiliations:** ^1^Department of Experimental and Clinical Medicine, University of Florence, Italy; ^2^University Hospital of Geneva, Geneva, Switzerland; ^3^University of Modena and Reggio Emilia, Modena, Italy; ^4^Unit of Internal Medicine, Department of Internal Medicine, SS Annunziata Hospital, Cento, Ferrara, Italy; ^5^“G. Fontana” Centre for the Study and Multidisciplinary Treatment of Alcohol Addiction, Department of Medical and Surgical Sciences, University of Bologna, Bologna, Italy; ^6^CIBER-EHD, Instituto de Salud Carlos III, Madrid, Spain; ^7^Hepatology and Liver Transplantation Unit, IIS La Fe, Hospital Universitario y Politécnico La Fe, Valencia, Spain; ^8^Faculty of Medicine, University of Valencia, Valencia, Spain; ^9^CIBERehd, Centro de Investigación Biomédica en Red en Enfermedades Hepáticas y Digestivas, Instituto de Salud Carlos III, Spain

In the article titled “Alcoholic and Nonalcoholic Liver Disease: Diagnostic Assessment and Therapeutic Perspectives” [1], the affiliation “University of Valencia, Hospital La Fe, Valencia, Spain” was incorrectly assigned to the author Dr. Marina Berenguer. The correct affiliations for this author are shown below, and they have been added as affiliations 6, 7, and 8 in the author information above:

CIBER-EHD, Instituto de Salud Carlos III, Madrid, Spain

Hepatology and Liver Transplantation Unit, IIS La Fe, Hospital Universitario y Politécnico La Fe, Valencia, Spain

Faculty of Medicine, University of Valencia, Valencia, Spain
